# Tuberculosis-HIV Co-infection in Kiev City, Ukraine

**DOI:** 10.3201/eid1205.051103

**Published:** 2006-05

**Authors:** Marieke J. van der Werf, Olga B. Yegorova, Nelly Chentsova, Yuriy Chechulin, Epco Hasker, Vasyl I. Petrenko, Jaap Veen, Leonid V. Turchenko

**Affiliations:** * KNCV Tuberculosis Foundation, The Hague, the Netherlands;; † Kiev City Tuberculosis Department, Kiev, Ukraine;; ‡ Kiev Anti-AIDS Centre, Kiev, Ukraine;; § Project Tuberculosis Prevention and Control in Kiev City, Ukraine, Kiev, Ukraine;; ¶ O.O. Bogmolic National Medical University, Kiev City, Ukraine

**Keywords:** HIV, tuberculosis, co-infection, risk factors, Ukraine, Eastern Europe

## Abstract

In 2004, we tested all patients with newly diagnosed tuberculosis (TB) for HIV in Kiev City. The results were compared to information from medical records of 2002, when co-infection prevalence was 6.3%. Of 968 TB patients, 98 (10.1%) were HIV infected. TB-HIV co-infection is increasing, especially in injecting drug users.

In Ukraine, the prevalence of HIV infection has been increasing since the mid-1990s (*1**,**2*). By January 2005, a total of 74,856 cases of HIV infection had officially been registered (http://www.aidsalliance.kiev.ua/). The real number of HIV infected persons may be much higher, an estimated 330,000–410,000 in 2001 (>1% of adult population) (*3*).

An increase in HIV prevalence is usually closely followed by an increase in tuberculosis (TB) (*4*). In Kiev City, the number of TB patients registered for treatment doubled from 629 in 1992 to 1,274 in 2004. This increase is mainly explained by economic and social changes after independence (August 1991), but the progressing HIV epidemic may also play a role in the increase in the number of TB patients.

In a previous study in Kiev City, we estimated the prevalence of HIV infection in patients with newly diagnosed TB at 6.3% in 2002 (*5*). In this study, we assess the prevalence of HIV infection ≈2 years later and compare the results of the 2 studies. We also determine risk factors for TB-HIV co-infection.

## The Study

From March 2004 to February 2005, all patients with newly diagnosed TB, who were >18 years of age and living in Kiev City, and who had begun anti-TB chemotherapy in the Kiev City TB Services were eligible for inclusion. Patients were informed about the study, counseled, and asked to participate. Basic information was collected about all TB patients from medical records and by interview. Reasons for not providing a blood sample for HIV testing were also recorded.

In Ukraine, TB diagnosis is made by smear and culture examination. All persons with suspected TB are evaluated by a committee of experts. TB is classified as pulmonary TB bacteriologically confirmed (smear or culture positive), pulmonary TB bacteriologically not confirmed (smear and culture negative or not done), and extrapulmonary TB.

Blood samples were tested for HIV by using Genscreen Plus HIV Ag-Ab (Bio-Rad Laboratories, Steenvoorde, France). Confirmation of the test result was done by Abbott IMx system HIV-1/-2 3rd Generation Plus (M/S Abbott GmbH, Wiesbaden, Germany). We tried to retest cases with an indeterminate HIV test result. TB patients with a positive test result were referred to the Kiev Anti-AIDS Centre.

We used SPSS 12.0 (SPSS Inc., Chicago, IL, USA) for data analysis with *t* tests and χ^2^ tests. Differences at the α = 5% level were regarded as significant. We examined predictive factors for HIV infection by logistic regression. The results were compared to those of a study using medical record information from patients newly diagnosed with TB in Kiev City in 2002 (*5*).

The study was approved by the medical ethics committee of the Yanovskiy Institute of Phtisiology and Pulmonology, Kiev City, Ukraine. Written informed consent was obtained from all participants.

A total of 1,090 TB patients were included from the 9 TB clinics and hospitals in Kiev City. Of those 1,090 TB patients, 4 (0.4%) could not be counseled because they were too ill or intellectually impaired, 83 (7.6%) did not provide informed consent, and 15 (1.4%) had to be excluded from the study, primarily because medical workers could not obtain a blood sample. The 102 (9.4%) TB patients who did not participate in the study were significantly older than those included: mean ages, respectively, 45.4 and 39.1 years (p<0.001). Other characteristics were not significantly different.

Of the 988 TB patients tested for HIV infection, 33 (3.3%) had an initial indeterminate test result. Sixteen of those were retested, 3 refused retesting, and 14 were not approached. Of those retested, 12 tested negative, 1 tested positive, and 3 again had an indeterminate test result. Thus, 968 TB patients with a definite HIV test result could be included in the analysis. Of those, 98 (10.1%) were HIV infected, 64 (65.3%) were identified in our study as HIV infected, and 34 (34.7%) had received a diagnosis of HIV infection from the Kiev Anti-AIDS Centre laboratory before being referred to the TB services with suspected TB.

Reported injecting drug use was the strongest independent predictor for HIV infection (Table 1). Those reporting injecting drug use were 31.4 times more likely to be HIV infected than those not reporting injecting drug use (95% confidence interval [CI] 17.4–56.9). Also, those who had reported a sexually transmitted disease in the past 5 years were more often HIV infected (odds ratio [OR] 4.4, 95% CI 1.6–12.4).

**Table 1:** Risk factors for a positive HIV test in patients with newly diagnosed TB in Kiev City, Ukraine*

Variable	No. (% HIV infected)	Univariate, OR (95% CI)	Multivariate, OR (95% CI)
Sex			
Male	712 (11.0)	1	
Female	256 (7.8)	0.69 (0.41–1.15)	
Age, y			
18–29	318 (14.8)	1	1
30–39	224 (17.4)	1.22 (0.76–1.93)	1.69 (0.94–3.04)
40–49	195 (4.6)	0.28 (0.13–0.58)	0.56 (0.24–1.30)
>50	231 (1.3)	0.08 (0.02–0.25)	0.18 (0.05–0.62)
Classification			
PTB+	541 (10.5)	1	
PTB-	379 (8.2)	0.76 (0.48–1.20)	
EPTB	48 (20.8)	2.24 (1.06–4.72)	
STD in last 5 y			
No	880 (8.6)	1	1
Yes	27 (22.2)	3.02 (1.18–7.72)	4.41 (1.57–12.38)
Unknown	61 (26.2)	3.76 (2.03–7.00)	1.99 (0.84–4.71)
Homeless			
Yes	56 (12.5)	1.29 (0.57–2.93)	
No	912 (10.0)	1	
Injecting drug use			
Yes	84 (66.7)	40.10 (23.15–69.45)	31.42 (17.35–56.87)
No	884 (4.8)	1	1
Abuse of alcohol			
Yes	105 (10.5)	1.04 (0.54–2.03)	
No	863 (10.1)	1	
Incarcerated >1994			
Yes	117 (15.1)	1.75 (1.01–3.05)	
No	851 (9.4)	1	

*n = 968; TB, tuberculosis; OR, odds ratio; CI, confidence interval; STD, sexually transmitted disease; PTB+, pulmonary TB bacteriologically confirmed; PTB–, pulmonary TB bacteriologically not confirmed; EPTB, extrapulmonary TB.

The prevalence of HIV infection among TB patients significantly increased from 6.3% in 2002 to 10.1% from March 2004 through February 2005 (p = 0.011) (Table 2). The prevalence of HIV-infected TB patients who reported injecting drug use increased from 1.8% of all tested patients with newly diagnosed TB in 2002 to 5.8% in March 2004 through February 2005. Thus, the main increase in TB-HIV co-infection was attributable to an increase in TB-HIV co-infected patients who reported injecting drug use. A larger proportion of persons with a positive HIV test result reported injecting drug use in 2004 (57.1%) than in 2002 (27.8%) (p = 0.003).

**Table 2:** Comparison of TB patients tested for HIV in 2002 and 2004

Variable	2002 study, n = 567 (%)	2004 study, n = 968 (%)	p
HIV infected			0.011
Yes	36 (6.3)	98 (10.1)	
No	531 (93.7)	870 (89.9)	
Sex			0.720
Male	412 (72.7)	712 (73.6)	
Female	155 (27.3)	256 (26.4)	
Age, y			0.091
0–29	153 (27.0)	318 (32.9)	
30–39	133 (23.5)	224 (23.1)	
40–49	131 (23.1)	195 (20.1)	
>50	150 (26.5)	231 (23.9)	
Classification*			0.002
PTB+	360 (63.5)	541 (55.9)	
PTB–	172 (30.3)	379 (39.2)	
EPTB	35 (6.2)	48 (5.0)	
Homeless			0.508
Yes	38 (6.7)	56 (5.8)	
No	529 (93.3)	912 (94.2)	
Injecting drug user			<0.001
Yes	16 (2.8)	84 (8.7)	
No	551 (97.2)	884 (91.3)	
Abuse of alcohol			0.020
Yes	85 (15.0)	105 (10.8)	
No	482 (85.0)	863 (89.2)	
Ever incarcerated			<0.001
Yes	40 (7.1)	132 (13.6)	
No	527 (92.9)	836 (86.4)	

*TB, tuberculosis; PTB+, pulmonary TB bacteriologically confirmed; PTB–, pulmonary TB bacteriologically not confirmed; EPTB, extrapulmonary TB.

HIV co-infection prevalence may be slightly overestimated in the 2002 study (*5*). In the study conducted between March 2004 and February 2005, TB patients included in the study were more frequently <50 years of age (p<0.001). TB patients <50 years of age were more frequently HIV infected. Both studies may therefore overestimate the prevalence of TB-HIV co-infection.

## Conclusions

HIV infection increased in patients with newly diagnosed TB in Kiev City between 2002 and 2004. This finding is in agreement with the increase in the number of registered cases of HIV infection in Ukraine since 1995 (*6*).

The main risk factor for being co-infected with HIV was reported injecting drug use. In 2002, 62.5% of the TB patients that reported injecting drug use were HIV infected and in 2004 this number was 66.7%. In Ukraine, the HIV epidemic started in injecting drug users thus that the main risk factor for HIV infection was injecting drug use is not surprising.

We used voluntary confidential HIV testing. Previous studies have found that use of this testing method can result in participation bias because those at higher risk of infection are more likely not to contribute specimens (*7**–**11*) or selection bias if clinicians encourage testing in those they consider to be more at risk (*5*). Although unlinked anonymous testing would have prevented these problems, the TB physicians participating in the study believed that using this strategy was not feasible. In our study, 7.6% refused to provide informed consent. This finding is comparable to researchers' experiences in other countries (*7**,**11**,**12*).

In the 2002 study, HIV testing was performed with a locally produced HIV test with unknown specificity and sensitivity. We do not know whether the estimated co-infection prevalence is valid or if we are likely to underestimate the true co-infection prevalence. Even if the locally produced HIV test did not correctly identify 12% of the HIV-positive patients (sensitivity 88%), the prevalence of TB-HIV co-infection was still significantly higher in 2004.

In the 2002 study, the percentage of persons not tested was high (38.0%), compared to a proportion of 10.5% in the 2004 study. If we assume that no HIV infections existed among those not tested, the minimum HIV-infection prevalence in the 2002 study is 3.9% and in the 2004 study 9.1%. No notable differences were identified between those tested and those not tested in variables that were strongly associated with HIV infection. In conclusion, TB-HIV co-infection is increasing in Kiev City, especially in injecting drug users.

## References

[1] Hamers FF. HIV infection in Ukraine (1987–96). Rev Epidemiol Sante Publique. 2000;48(Suppl 1):1S3–15. 10854992

[2] Hamers FF, Downs AM. HIV in central and eastern Europe. Lancet. 2003;361:1035–44. 10.1016/S0140-6736(03)12831-0 12660072

[3] Balakireva O, Galustian Y, Yaremenko O, Scherbyns'ka A, Kruglov Y, Levchuk N, The social and economic impact of HIV and AIDS in Ukraine: a re-study. Kyiv City; Ukraine: British Council; 2001.

[4] Corbett EL, Watt CJ, Walker N, Maher D, Williams BG, Raviglione MC, The growing burden of tuberculosis: global trends and interactions with the HIV epidemic. Arch Intern Med. 2003;163:1009–21. 10.1001/archinte.163.9.1009 12742798

[5] van der Werf MJ, Yegorova OB, Chechulin Y, Hasker E, Veen J, Turchenko LV. HIV testing practices of TB patients after introduction of a new testing policy in Kiev City, Ukraine. Int J Tuberc Lung Dis. 2005;9:733–9. 16013767

[6] Mavrov GI, Bondarenko GM. The evolution of sexually transmitted infections in the Ukraine. Sex Transm Infect. 2002;78:219–21. 10.1136/sti.78.3.219 12238659 PMC1744475

[7] Hull HF, Bettinger CJ, Gallaher MM, Keller NM, Wilson J, Mertz GJ. Comparison of HIV-antibody prevalence in patients consenting to and declining HIV-antibody testing in an STD clinic. JAMA. 1988;260:935–8. 10.1001/jama.1988.03410070063029 3398198

[8] Jones JL, Hutto P, Meyer P, Dowda H, Gamble WB Jr, Gunn RA. HIV seroprevalence and reasons for refusing and accepting HIV testing. Sex Transm Dis. 1993;20:334–7. 10.1097/00007435-199320060-00006 8108756

[9] Groseclose SL, Erickson B, Quinn TC, Glasser D, Campbell CH, Hook EW III. Characterization of patients accepting and refusing routine, voluntary HIV antibody testing in public sexually transmitted disease clinics. Sex Transm Dis. 1994;21:31–5. 10.1097/00007435-199401000-00007 8140486

[10] Postema EJ, Willems PW, de Ridder MA, van der Meijden WI. Comparison of patients refusing with patients accepting unlinked anonymous HIV testing in an outpatient STD department in The Netherlands. Int J STD AIDS. 1997;8:368–72. 10.1258/0956462971920271 9179646

[11] Paget WJ, Zwahlen M, Eichmann AR. Voluntary confidential HIV testing of STD patients in Switzerland, 1990–5: HIV test refusers cause different biases on HIV prevalences in heterosexuals and homo/bisexuals. Swiss Network of Dermatovenereology Policlinics. Genitourin Med. 1997;73:444–7. 9582457 10.1136/sti.73.6.444 PMC1195921

[12] Abouya L, Coulibaly IM, Wiktor SZ, Coulibaly D, N'Krogbo M, N'Gbo A, The Côte d'Ivoire national HIV counseling and testing program for tuberculosis patients: implementation and analysis of epidemiologic data. AIDS. 1998;12:505–12. 10.1097/00002030-199805000-00012 9543449

